# Topical netarsudil for the treatment of primary corneal endothelial degeneration in dogs

**DOI:** 10.1038/s41598-024-56084-4

**Published:** 2024-03-14

**Authors:** M. Isabel Casanova, Sangwan Park, Melaney A. Mayes, Karolina Roszak, Michelle Ferneding, Nayeli Echeverria, Morgan A. W. Bowman, Sarah R. Michalak, Monica Ardon, Sydni Wong, Sophie M. Le, Nicole Daley, Brian C. Leonard, Kathryn L. Good, Jennifer Y. Li, Sara M. Thomasy

**Affiliations:** 1 Department of Surgical and Radiological Sciences, School of Veterinary Medicine, University of California, 1 Shields Ave., Davis, CA 95616 USA; 2 William R. Pritchard Veterinary Medical Teaching Hospital, School of Veterinary Medicine, University of California Davis, Davis, CA 95161 USA; 3 Department of Ophthalmology and Vision Science, School of Medicine, University of California Davis, Davis, CA 95616 USA

**Keywords:** Eye diseases, Translational research

## Abstract

This study evaluated the tolerability and efficacy of the topical rho-kinase inhibitor netarsudil for canine primary corneal endothelial degeneration (PCED). Twenty-six eyes of 21 client-owned dogs with PCED were enrolled in a prospective, randomized, vehicle control clinical trial and received topical netarsudil 0.02% (Rhopressa®) or vehicle control twice daily (BID) for the first 4 months. Then, all patients received netarsudil for the next 4 or 8 months. Complete ophthalmic examination, ultrasonic pachymetry*,* Fourier-domain optical coherence tomography, and in vivo confocal microscopy were performed at baseline and 1, 2, 4, 6, 8 and 12 months. Effect of netarsudil on central corneal thickness (CCT), percentage of cornea with edema, and endothelial cell density (ECD) were evaluated by repeated measures ANOVA. Kaplan–Meier curves and log-rank test were used to compare corneal edema and clinical progression of eyes in netarsudil versus vehicle control groups. All dogs developed conjunctival hyperemia in at least one eye while receiving netarsudil. Unilateral transient reticulated intraepithelial bullae and stromal hemorrhage were observed respectively in 2 dogs in the netarsudil group. Two dogs showed persistently decreased tear production while receiving netarsudil, requiring topical immunomodulatory treatment. No significant differences in CCT, ECD, corneal edema or clinical progression were observed between netarsudil or vehicle treated eyes. When comparing efficacy of topical netarsudil BID and topical ripasudil 0.4% administered four times daily from our previous study, dogs receiving ripasudil had significantly less progression than those receiving netarsudil.

## Introduction

The corneal endothelium is responsible for the maintenance of stromal dehydration by active transport of ions using Na^+^/K^+^ ATPase pumps^1^. Canine corneal endothelial cells have a limited regenerative capacity^2^, thus, their loss can lead to functional decompensation and corneal edema^1^. Corneal endothelial dystrophy and age-related endothelial degeneration are two common causes of primary corneal endothelial degeneration (PCED)^3,4^. In humans, the most common endothelial degenerative disease is Fuchs’ endothelial corneal dystrophy (FECD), which is one of the leading indications for penetrating and endothelial keratoplasty in humans^5,6^. These surgical techniques also improve corneal edema in canine patients with corneal endothelial dystrophy^7,8^. However, the lack of donor tissue, high cost of corneal surgery, and risk of complications are major limitations to the implementation of these surgical procedures in veterinary medicine, and they are rarely performed in canine patients. Instead, palliative therapies are utilized to relieve the clinical signs^9^. Topical application of sodium chloride 5% ophthalmic ointment has been employed to reduce or delay corneal edema progression^10^, despite limited efficacy at reducing corneal thickness in normal dogs^11^. Palliative surgical procedures such as superficial keratectomy and conjunctival advancement hood flap (SKCAHF) may be advised in some dogs in advanced stage of disease^12^. However, these surgical treatments are invasive, entail surgical and anesthetic risks, and the effect is temporary, which are limitations that must be taken into consideration.

The Rho-associated coiled-coil kinases (ROCK) 1 and 2 are widely distributed in the mammalian body and have multiple functions, including regulation of the cytoskeleton, cellular contraction and cellular motility, and regulation of cellular morphology, polarity, apoptosis, and cell division^13^. In vitro studies have shown that activation of the Rho/Rho kinase pathway is involved in corneal endothelial cell apoptosis^14^, and that ROCK inhibitors promote corneal endothelial cell survival and adhesion^15^. In vivo studies in rabbit^16^, dogs^17^, and non-human primates (NHP)^18^ employing cryoinjury models have shown that topical ROCK inhibitors accelerate corneal endothelial regeneration after corneal endothelial wounding^16,18^. A pilot study in humans reported improvement in corneal clarity and vision following administration of topical netarsudil 0.02% ophthalmic solution administration once daily for 3 months in patients with FECD^19^. Furthermore, injection of corneal endothelial cells in combination with ROCK inhibitors have restored corneal clarity and improved vision in humans with bullous keratopathy^20^. Finally, the administration of ROCK inhibitors facilitated a faster recovery in vision in human patients with endothelial disease undergoing Descemet stripping surgery^21,22^.

Based on these promising data, we recently completed a prospective, open-label clinical trial to determine the safety and efficacy of 0.4% ripasudil (Glanatec®) as a treatment for canine PCED. This study demonstrated stabilization or improvement of clinical disease in 62% of eyes included in the study after one year of treatment^23^. A more favorable response was found in canine patients at an early disease stage^23^. However, ripasudil is not commercially available in the US and requires 4 times a day (QID) application. By contrast, 0.02% netarsudil (Rhopressa®) is an FDA-approved ROCK inhibitor and norepinephrine transporter inhibitor for the treatment of glaucoma in humans that is typically administered once or twice a day (BID). The purpose of our study was to perform a prospective, double-masked, vehicle controlled clinical trial to evaluate the tolerability and efficacy of netarsudil administered BID for the treatment of canine PCED.

## Results

**Figure 1:**
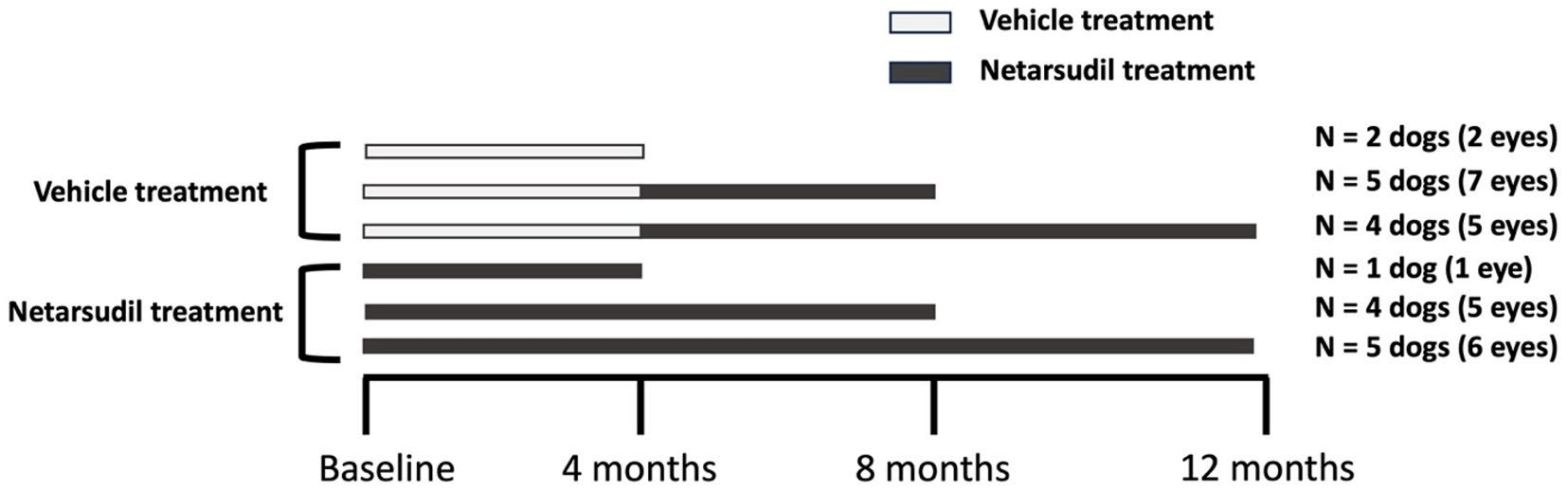
Study design depicting allocation of dogs to groups and their duration of treatment. Canine patients were divided into vehicle treatment group (n = 11) and netarsudil treatment group (n = 10). Of the 11 dogs enrolled in the vehicle treatment group, 2 dogs were treated for 4 months with vehicle only, 5 dogs were treated for 4 months with vehicle then 4 months with netarsudil, and 4 dogs were treated for 4 months with vehicle then 8 months with netarsudil. Of the 10 dogs enrolled in the netarsudil treatment group, 1, 4 and 5 dogs were treated for 4, 8 and 5 months, respectively.

Twenty six eyes of 21 PCED-affected dogs were included in this study. Ten dogs were allocated to receive netarsudil while eleven were assigned to receive vehicle (Fig. 1). The study population consisted of 7 Boston terriers, 3 Chihuahua mixed breed dogs, 3 Jack Russell terriers, 2 boxers, 1 Labrador retriever, 1 basset hound, 1 standard poodle, 1 shih tzu mixed breed, 1 Schipperke and 1 Brittany (Supplementary Table 1). Mean age at enrollment was 10.9 ± 0.9 and 10.6 ± 2.7 years in the netarsudil and vehicle control groups, respectively, and did not significantly differ (*P* = 0.74*)*. Four males and 6 females were enrolled in the netarsudil group and 4 males and 7 females were enrolled in the vehicle control group (*P* > 0.99). Of the 21 dogs included in the study, 13 (7 Boston terrier, 3 Jack Russell terriers and 3 Chihuahua mix dogs) were diagnosed with corneal endothelial dystrophy, while the remaining 8 dogs were diagnosed with age-related endothelial degeneration. Of the ten dogs in the netarsudil group, eight received unilateral treatment and two received bilateral treatment. Five patients (6 eyes) from the netarsudil group were treated for a total of 12 months. One patient was lost to follow up 5 months after enrollment in the study (Fig. 1).

In the vehicle control group, 8 dogs were treated unilaterally, and 3 dogs were treated bilaterally. All patients received vehicle during the first 4 months. Afterwards, 5 patients received netarsudil for 4 months and 4 (5 eyes) patients received netarsudil for 8 months. Two patients in this group were humanely euthanized for reasons unrelated to ocular disease 5 and 7 months after enrollment in the study, respectively (Fig. 1).

**Figure 2:**
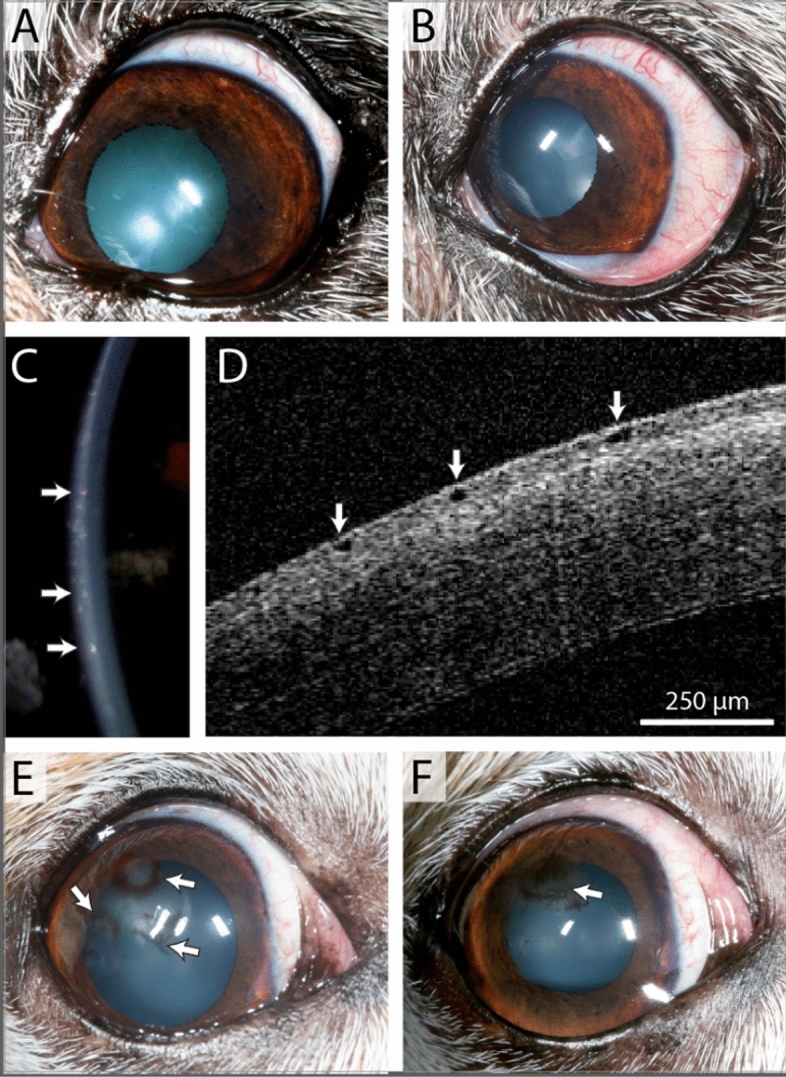
Adverse events observed in a 10-year-old female Jack Russell terrier (**A–D**) and a 12-year-old Chihuahua mix (**E, F**) receiving topical netarsudil twice daily. Mild increase in conjunctival hyperemia developed after 4 months of topical netarsudil application (**B**) in comparison to before starting topical netarsudil (**A**). With slit lamp (**C**) and FD-OCT (**D**), intraepithelial bullae were observed 1 month after starting topical netarsudil (arrows). Focal corneal stromal hemorrhage associated with corneal vascularization was identified at 8 months (**E**). This dog had received topical netarsudil twice daily for the entire study period. The hemorrhage resolved 1 month after discontinuation of topical netarsudil and 4 months later, subepithelial melanin was observed in the superotemporal paraxial cornea (**F**).

The most common adverse reaction observed with netarsudil treatment was conjunctival hyperemia, which was recorded at least once during ophthalmic examination in all dogs in at least one timepoint while receiving topical netarsudil (Supplementary Table 1). However, even although the medication was not discontinued, no dogs required treatment for the conjunctival hyperemia. Reticulated intraepithelial bullae were observed in one eye receiving topical netarsudil of one dog at a single timepoint that resolved without discontinuing the medication. One patient with corneal melanosis and vascularization that was receiving cyclosporine 0.2% ophthalmic ointment (Optimmune®, USP, Merk & Co, Kenilworth, NJ) developed corneal stromal hemorrhage in one eye after 8 months of netarsudil treatment. After discontinuation of topical netarsudil, the hemorrhage resolved in 1 month. The corneal melanosis slightly progressed in the eye of this patient (Fig. 2).

**Figure 3:**
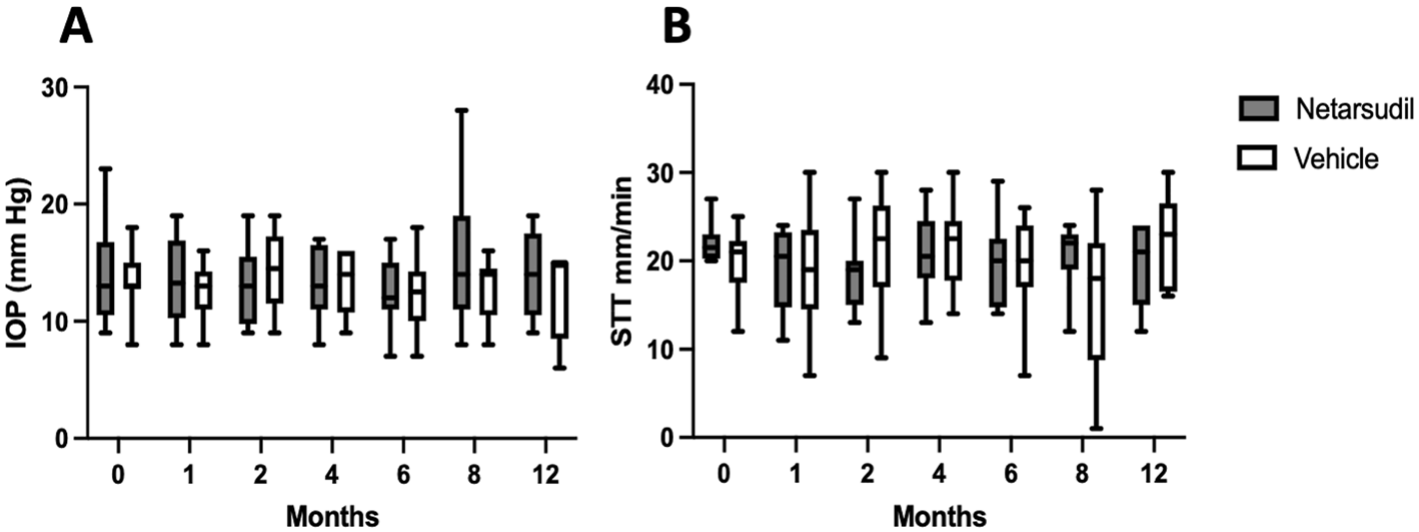
No significant differences were observed in IOP (**A**) or STT-1 values (**B**) between the netarsudil (10 dogs, 12 eyes) and vehicle control (11 dogs, 14 eyes) groups or baseline and any timepoints. Box plots depict median, and 25th and 75th percentiles. Whisker plots show maximum and minimum values. Two-way repeated measures ANOVA was performed. No differences in STT or IOP were observed between netarsudil and vehicle control groups at any timepoint or in comparison to baseline, *P* > 0.05. IOP: intraocular pressure as measured by rebound tonometry. STT: Schirmer tear test. Netarsudil group: baseline, 1, 2 and 4 months: 12 eyes; Six and 8 months: 11 eyes; 12 months: 6 eyes. Vehicle group: baseline, 1, 2 and 4 months: 14 eyes; 6 months: 13 eyes; 8 months: 12 eyes; 12 months: 5 eyes.

No differences in STT or IOP were observed between netarsudil and vehicle control groups at any timepoint or in comparison to baseline (Fig. 3). Of the 14 dogs included in this study, two were presented with decreased tear production while receiving topical netarsudil that required cyclosporine 0.2% ophthalmic ointment (Optimmune®) in one (n = 1) or both (n = 1) eyes with a frequency that ranged from every other day to BID. One patient had an IOP decrease from 17 to 10 mm Hg 1 week after initiating topical netarsudil; no signs of ocular inflammation were observed. After receiving ketorolac tromethamine 0.5% ophthalmic solution once daily for a week, the IOP returned to a normal value (14 mm Hg) and remained at that pressure over the rest of the study; the ketorolac was discontinued after a week of treatment. One patient developed ocular hypertension in one eye (28 mm Hg) 6 months after starting the topical netarsudil that was managed with dorzolamide HCl 2% ophthalmic solution applied 3 times a day during the next 2 months. The IOP was then stable (21 mm Hg) and the frequency of topical dorzolamide was reduced to BID for the remainder of the study.

**Figure 4:**
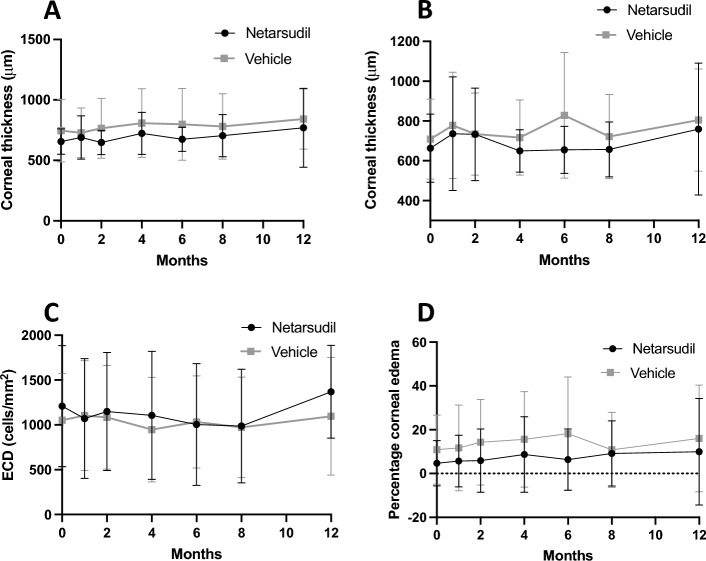
No significant differences were observed in CCT measured by USP (**A**), corneal stromal thickness measured by OCT (**B**), ECD measured with non-applanation IVCM (**C**), and percentage of cornea affected by edema (**D**) in patients treated with netarsudil during 8 months versus patients receiving vehicle control during the first 4 months. Two-way repeated measures ANOVA. Depicted are the mean and the standard deviation. For CCT, USP, percentage of corneal edema: Netarsudil group: baseline, 1, 2 and 4 months: 12 eyes. Six months and 8 months: 11 eyes. Twelve months: 6 eyes. Vehicle group: baseline, 1, 2 and 4 months: 14 eyes. Six months: 13 eyes. Eight months: 12 eyes. Twelve months: 5 eyes. ECD: Netarsudil group: baseline, 1, 2 and 4 months: 12 eyes. Six and 8 months: 11 eyes. Twelve months: 5 eyes. Vehicle group: baseline, 1, 2 and 4 months: 14 eyes. Six months: 13 eyes. Eight months: 12 eyes. Twelve months: 3 eyes.

No significant differences were observed in CCT measured at baseline with USP between the netarsudil (mean 656 ± 106 μm) and the vehicle control (mean 745 ± 258 μm) groups (*P* = 0.82). There were no differences in CCT between baseline and any timepoint post-treatment for either netarsudil or vehicle control groups (*P* > 0.05). Additionally, CCT did not significantly differ between the netarsudil and the vehicle control groups at any timepoint post-treatment (*P* > 0.05, Fig. 4a). Differences in corneal thickness measured with USP at the nasal, temporal, dorsal and ventral cornea also did not differ between baseline and any timepoint post-treatment for either netarsudil or vehicle control (*P* > 0.05) nor between netarsudil and vehicle control groups at any timepoint post-treatment (*P* > 0.05, Supplementary Fig. 1). Median [interquartile range, IQR] corneal stromal thickness measured with FD-OCT at baseline did not significantly differ between the netarsudil (524 [464–592] μm) and the vehicle control groups (586 [493–698] μm *P* = 0.84). There were no significant differences in stromal thickness at any time post-treatment (*P* > 0.05), nor between groups at any timepoint (*P* > 0.05, Fig. 4b).

At baseline, mean ECD when measured with non-applanation IVCM did not significantly differ at 1236 ± 671 cells/mm^2^ and 1052 ± 522 in the netarsudil and vehicle control groups, respectively (*P* = 0.97). No significant differences were observed in central ECD between baseline and any timepoint post-treatment for either netarsudil or vehicle control groups (*P* > 0.05) nor between the netarsudil and the vehicle control groups at any time point (*P* > 0.05, Fig. 4c). Similarly, there were no differences in ECD measured with applanation IVCM between baseline and any timepoint post-treatment for either netarsudil or vehicle control treatments (*P* > 0.05) nor between the vehicle control and the netarsudil group at any time point (*P* > 0.05, Supplementary Fig. 2).

Of the 26 eyes included in this study, 3 eyes from three dogs in the netarsudil group and 5 eyes from five dogs in the vehicle control group had corneal edema at baseline; the remaining 18 eyes had clear corneas During the study, 4 eyes from four dogs (2 in the netarsudil group, 2 in the vehicle control group) developed corneal edema. In this study, no significant differences were observed in percentage of corneal edema in each group during the study (*P* > 0.05), neither between groups at any time point (*P* > 0.05, Fig. 4d).

**Figure 5:**
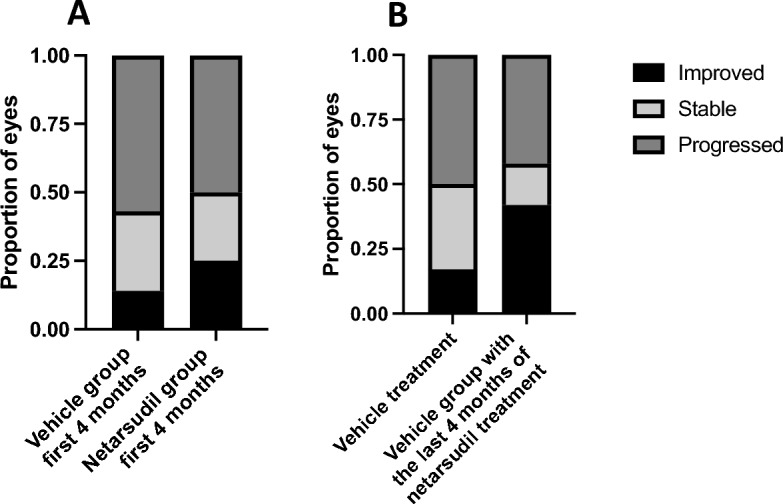
Clinical improvement or stabilization of clinical disease did not significantly differ for canine patients receiving topical netarsudil versus those receiving vehicle. There was no significant correlation between the proportion of eyes that were improved or remained stable on patients in the netarsudil group that received netarsudil for 4 months when compared with eyes in the vehicle control group at 4 months (**A**, *P* = 0.77 Fisher exact test, Freeman-Halton extension). Similarly, there was no significant difference in progression in the vehicle control group during the first 4 months of vehicle treatment versus the last 4 months with netarsudil treatment (**B**, *P* = 0.35, Fisher exact test, Freeman-Halton extension).

**Figure 6:**
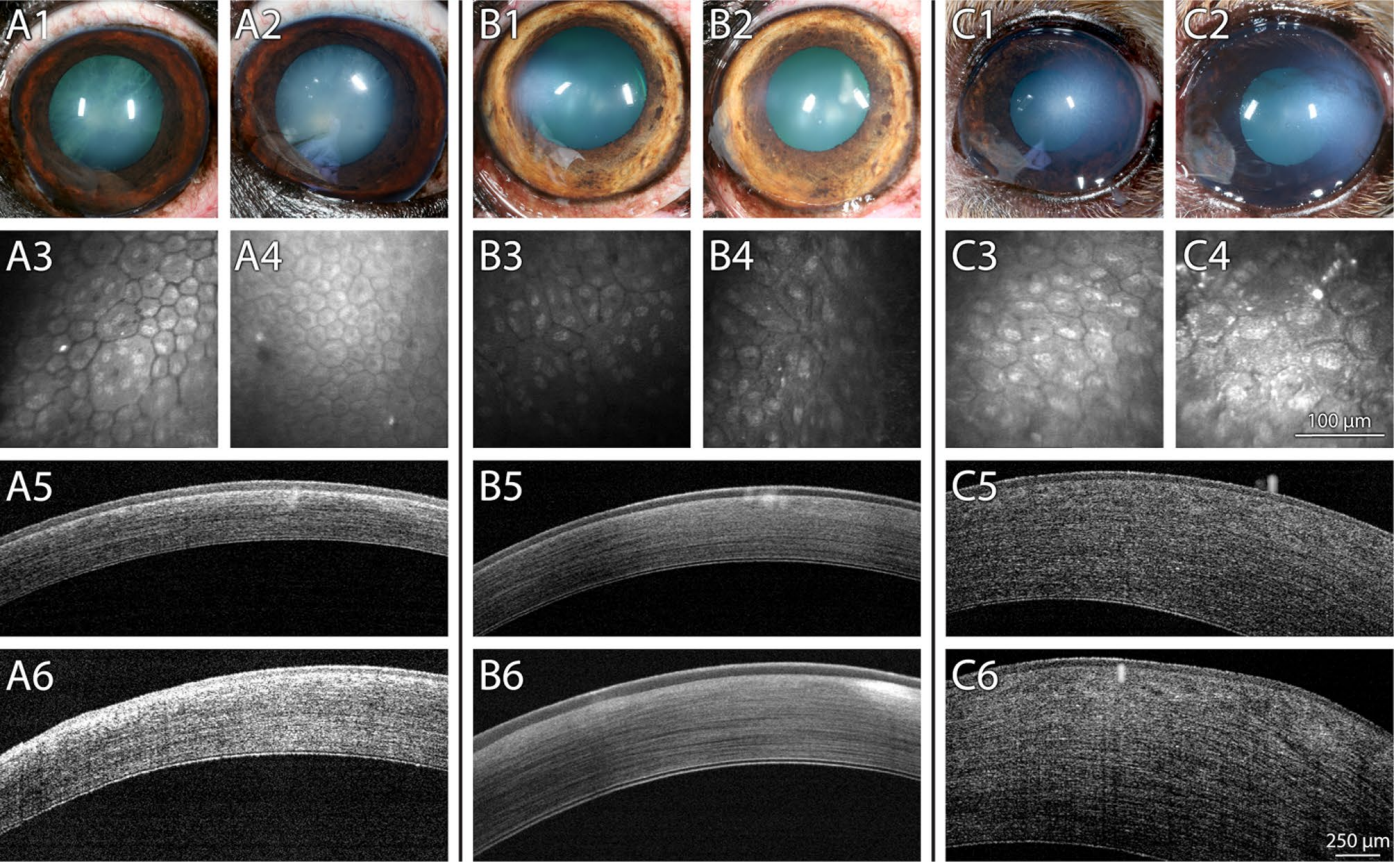
Dogs treated with topical netarsudil BID for 8 months demonstrated variable disease progression. (**A**) Left eye of a 11-year-old neutered male Boston terrier diagnosed with PCED that received topical netarsudil for 8 months. The corneal edema was stable during the study (A1, A2) and the ECD increased from 944 ± 443 cells/mm^2^ (A3) to 1978 ± 123 cells/mm^2^ (A4). Corneal thickness remained stable (A5—575 μm, A6—624 μm). (**B**) Right eye of a 9.5-year-old neutered male boxer diagnosed with PCED that received topical netarsudil for 8 months. The area of the cornea affected by edema was stable during the study with 12.3% of the cornea affected by edema at baseline (B1) and 7% after 8 months of treatment (B2), as well as ECD (356 ± 107 cells/mm^2^ at baseline, B3, and 389 ± 212 cells/mm^2^ after 8 months, B4) and CCT (695 μm at baseline, B5, and 662 μm after 8 months, B6). (**C**) Left eye of a 11-year-old spayed female Chihuahua mix diagnosed with PCED that received topical netarsudil for 8 months. While the area of the cornea affected by edema remained stable during the study (34.8% at baseline, C1, and 41.3% after 8 months, C2), there was a decrease in ECD from 856 ± 175 cells/mm^2^ (C3) to 478 ± 32 cells/mm^2^ (C4). Central corneal thickness increased from 790 μm (C5) to 1171 μm (C6). Bar on IVCM images = 100 μm. Bar on FD-OCT images = 250 μm.

**Figure 7:**
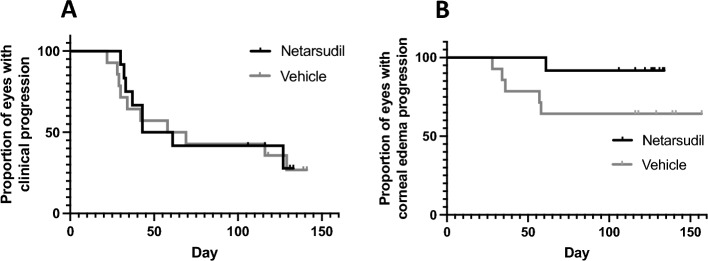
Kaplan Meier curves demonstrate no significant difference in clinical progression using multiple criteria (**A**) or corneal edema progression (**B**) in canine PCED patients treated with netarsudil or vehicle control for the initial 4 months. Using the combined clinical criteria (**A**) or only corneal edema progression (**B**), no statistical differences were observed between patients treated with netarsudil and patients treated with vehicle control during the first 4 months (**A**, *P* = 0.84; **B**, *P* = 0.08). For the Kaplan Meier curves, progression was established using combined clinical criteria (**A**, progressed if eyes demonstrated a > 10% decrease in ECD, a > 10% increase of cornea affected by edema, and/or a > 20% increase in CCT) and corneal edema progression alone (**B**, when the eye had an increase of > 10% in area of the cornea affected by edema).

We next evaluated disease progression using the combined clinical response criteria. During the first 4 months, 3 eyes improved, 3 eyes were stable, and 6 progressed in the netarsudil group while 2 eyes improved, 4 were stable, and 8 progressed in the vehicle control group (Fig. 5a). Twelve eyes remained in the trial after the vehicle control phase and received netarsudil for the next 4 months. Of those, 5 eyes improved, 2 eyes remained stable, and 5 eyes progressed while receiving netarsudil (Fig. 5b). Out of the 11 eyes in the netarsudil group that received netarsudil for 8 months, when compared with baseline, 1 eye improved, 1 eye remained stable and 9 eye progressed. A direct association between netarsudil treatment and improvement or clinical disease stabilization was not evident (*P* > 0.05), and variable response to treatment was observed in the netarsudil group (Fig. 6). Kaplan–Meier curves and log rank test also demonstrated no statistically significant differences between patients in netarsudil and vehicle control groups when using the combined clinical criteria (Fig. 7a) and when using only corneal edema progression (Fig. 7b). Six eyes received netarsudil for 1 year and five eyes, initially allocated to the vehicle control group, received netarsudil for 8 months. Using the combined clinical criteria, when compared with baseline, only one of the 6 eyes that received netarsudil for 12 months improved, and one remained stable. In the vehicle control group, when compared with the start of netarsudil treatment, only 2 of 5 eyes that received 8 months of netarsudil remained stable and none improved.

**Figure 8:**
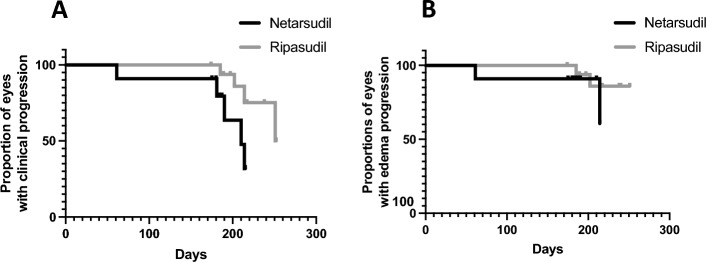
Kaplan Meier curves comparing clinical progression using the combined clinical criteria (**A**) and only corneal edema progression (**B**) in patients treated with ripasudil and netarsudil for 6 months. When looking at combined criteria, patients receiving ripasudil for 6 months were significantly less likely to progress compared with patients receiving netarsudil for 6 months (*P* = 0.04). However, no significant difference was observed when evaluating corneal edema only (*P* = 0.37). For these comparisons, data from 17 eyes from 11 dogs in treatment with ripasudil and 11 eyes from 9 dogs in treatment with netarsudil for 6-months were included. For the Kaplan Meier curves, progression was established using combined clinical criteria (**A**, progressed if eyes demonstrated a > 10% decrease in ECD, a > 10% increase of cornea affected by edema, and/or a > 20% increase in CCT) and corneal edema progression alone (**B**, when the eye had an increase of > 10% in area of the cornea affected by edema).

We next compared clinical progression of patients diagnosed with PECD receiving ripasudil QID^23^ (17 eyes from 11 dogs) and patients receiving netarsudil BID (11 eyes from 9 dogs) for 6 months using the combined clinical criteria and corneal edema progression. Dogs receiving ripasudil had significantly lower disease progression using the combed criteria in comparison to those receiving netarsudil (*P* = 0.04, Fig. 8a) although no significant difference was observed using only corneal edema progression (*P* = 0.37, Fig. 8b).

## Discussion

The purpose of this study was to evaluate the safety and efficacy of topical netarsudil BID application as a non-surgical alternative therapy to stabilize or slow disease progression in PCED-affected patients with < 40% of the cornea affected by edema. The results of this study indicate that netarsudil is well-tolerated in dogs, however, an obvious therapeutic effect for PCED was not observed when comparing the vehicle control and the netarsudil groups after 4 months of treatment.

In this clinical trial, we confirmed that similar to previous studies of topical ROCK inhibitors in dogs^23,24^, topical netarsudil BID application is well-tolerated, with conjunctival hyperemia being the most common adverse reaction. In our study, one patient experienced transient reticulated intraepithelial edema, which is an adverse reaction described in humans and dogs following treatment with ROCK inhibitors^23,25^. Another patient developed a corneal stromal hemorrhage by the end of the study, which has also been reported in human and canine patients receiving ROCK inhibitors^23,26^. Other adverse reactions described in human patients receiving topical netarsudil such as corneal verticillata were not observed in this study^27^. Two dogs were diagnosed with dry eye and required topical immunomodulatory treatment. A review of the literature did not identify any known association between ROCK inhibitors and alterations in tear production in humans or dogs. One possibility may be that the patients in this study developed age-related dry eye, as aging is a risk factor for dry eye disease in dogs^28^, but a potential association between topical netarsudil and dry eye cannot be excluded. As such, we recommend monitoring tear production in canine patients receiving topical netarsudil.

Due to their IOP-lowering effects, ROCK inhibitors are used to treat glaucoma in humans^27^. Consistent with previous canine studies^23,29^, IOP did not significantly differ from baseline or between groups. However, one eye of one dog was presented with a decrease in IOP in which treatment with anti-inflammatory medication for 1 week resulted in normalization of IOP. Thus, monitoring of IOP in canine patients receiving topical netarsudil is recommended.

Our study, using 21 dogs and our clinical criteria, did not identify a significant improvement in CCT, corneal stromal thickness, ECD, or corneal edema associated with netarsudil treatment. By contrast, our previous study with another ROCK inhibitor, ripasudil, demonstrated a more favorable response in canine PCED patients in early stage of disease, with 8/11 eyes (62%) classified as clinically stable or improved 1 year after starting topical ripasudil^23^. In comparison, only 9 of 23 eyes (39%) remained stable or improved following either 8 or 4 months of netarsudil treatment.

Cell-to-cell contact inhibition is an anti-proliferative regulatory mechanism, present in corneal endothelial cells^30^. In humans, removal of a portion of damaged corneal endothelium can stimulate proliferation and migration of the adjacent healthy endothelial cells, and the effect is thought to be boosted with the addition of ROCK inhibitors^31^. Indeed, ROCK inhibitors have been shown to facilitate a faster recovery of corneal transparency in FECD patients undergoing surgical procedures or transcorneal freezing^18,22^. Similarly, our previous study employing a canine model with healthy endothelium showed significant reduction in CCT and increase in ECD after cryoinjury in eyes treated with the ROCK inhibitor Y27632 when comparing with the vehicle control group^17^; however, the potential benefit of a combined therapy in canine patients with PCED is still unknown. Future studies to test the synergic effectivity of combining ROCK inhibitors and corneal surgical interventions in canine PCED patients are warranted.

When conducting a double-masked, prospective clinical trial, there are certain limitations that need to be acknowledged. Three of our 21 patients did not complete the trial either because they were humanely euthanized for reasons unrelated to the study (2) or were lost to follow up (1). The incorporation of sodium chloride hypertonic ophthalmic ointment as regular treatment for some patients during the trial might have impacted corneal edema and corneal thickness values since this medication can reduce corneal edema and thus corneal thickness^9,11^. Our study included data from eyes at different stages of clinical PCED, ranging from compensated stage at baseline (absence of corneal edema, 18 eyes) to up to 40% of the cornea affected by corneal edema (8 eyes). The variation of the clinical stage at the initiation of netarsudil may impact the efficacy of this treatment at reducing the progression of PCED. Furthermore, progressive or severe corneal edema prevented visualization of corneal endothelial cells with IVCM for some patients at various timepoints. Finally, the lack of progression in 7/12 eyes (58%) in the vehicle control group after 4 months of netarsudil treatment suggests that a treatment period longer than 4 months may be required to identify progression in CED patients with early disease.

## Conclusion

Topical netarsudil is well tolerated in PCED-affected canine patients, as the most common adverse reaction, conjunctival hyperemia, did not require medical treatment. In our study, no significant differences were observed between netarsudil and vehicle control groups for corneal thickness, ECD, or percentage of the cornea affected by edema indicating that its efficacy in canine PCED is limited. Ripasudil may be superior to netarsudil at delaying disease progression in dogs with PCED.

## Methods

### Animals

Dogs included in this study were exclusively client-owned. This study was approved by the Institutional Animal Care and Use Committee of the University of California-Davis and was in concordance with the Association for Research in Vision and Ophthalmology resolution on the use of animals in research, as well as the ARRIVE guidelines. Dogs with a presumptive diagnosis of PCED assigned by board-certified veterinary ophthalmologists from the University of California-Davis Veterinary Medical Teaching Hospital and other specialty hospitals in California and Nevada between March 2020 and October 2021 were included in this study. Dogs diagnosed with diseases that could cause secondary corneal endothelial cell injury such as glaucoma, intraocular surgery, lens instability, diabetes, and endotheliitis were excluded^32–35^, and only eyes with < 40% of the cornea affected by edema were included in the study. Prior to enrollment, informed consent was obtained for all dogs.

### Treatment

During the first 4 months, study patients were randomly assigned into netarsudil or vehicle control groups, and either received one drop of netarsudil 0.02% ophthalmic solution (Rhopressa®, Aerie Pharmaceuticals, NY, USA) or vehicle control (Rhopressa® vehicle) BID. Owners and clinicians were masked to the treatment during the first 4 months to objectively evaluate potential adverse reactions and benefits of receiving topical netarsudil. To evaluate long-term effects of the medication and as an incentive for owners, all dogs then received topical netarsudil for the next 4 additional months.

Owners recorded in a drug log form the time of application of the medication during the study. Additional medications, such as hypertonic saline ophthalmic ointment for the treatment of corneal edema and/or bullous keratopathy or any other ophthalmic medications, were prescribed when needed and noted for all patients.

Two patients were humanely euthanized at their local veterinary clinic by an overdose of intravenous pentobarbital (> 100 mg/kg) at 5 and 7 months after enrollment for reasons unrelated to the study.

### Ophthalmic examination and imaging

Prior to enrollment, all patients received a complete, ophthalmic examination to confirm the diagnosis of PCED. The exam included a Schirmer tear-test 1 (STT1; Intervet, Inc., Summit, NJ, USA), intraocular pressure (IOP) measurement by rebound tonometry (TonoVet; Icare® Finland), handheld and digital slit-lamp biomicroscopy (SL-15; Kowa American Corporation, Torrance, CA, USA and Imaging Module IM 900; Haag Streit, Koeniz, Switzerland), and binocular indirect ophthalmoscopy (Keeler Instruments Inc., Broomall, PA, USA) using a 28 diopter (D) indirect lens (Volk Optical, Inc., Mentor, OH, USA). Color photography (Canon OD 5S) with ImageJ software^36^ were employed to measure the area of the cornea affected by edema, as previously described^32^.

Central corneal thickness (CCT) and nasal, temporal, superior and inferior corneal thicknesses were measured using ultrasonic pachymetry (USP, Pachette 3; DGH Technology, Inc., Exton, PA, USA). Fourier-domain optical coherence tomography (FD-OCT, RTVue 100, software version 6.1, 26,000 A scan/sec, 5-μm axial resolution, 840-nm superluminescent diode, Optovue, Inc., Fremont, CA, USA) was employed to identify the different layers of the central cornea and manually measure their thickness, as previously described^32,37^. In vivo confocal microscopy (IVCM) of the central and medial cornea (ConfoScan 4, Nidek Technologies, Gamagori, Japan and ConfoScan 4 NAVIS imaging software) and of the central cornea (HRT III with Rostock Cornea Module, Heidelberg Engineering GmbH, Dossenheim, Germany) were performed to evaluate corneal endothelial cell morphology as described previously^32,37,38^. The corneal endothelial cell density (ECD) was calculated from IVCM scans following a system previously reported^23,39^. For ECD calculations, as well as for measurement by FD-OCT of the different layers of the cornea, the individual analyzing the images was masked for the group of treatment, timepoint, and identity of the patient. The image analysts (MAM, MIC) were masked for the group of treatment, timepoint, and identity of the patient.

Lastly, corneas were stained with fluorescein sodium (Ful-Glo strips USP 1 mg; Akorn Inc) to assess for corneal ulceration. Intravenous sedation with acepromazine (0.005–0.02 mg/kg) and butorphanol (0.1–0.3 mg/kg) or dexmedetomidine (1–3 µg/kg) was administered as needed for imaging. At the completion of the exam, intramuscular atipamezole (1–3 µg/kg) was administered as reversal for dogs receiving dexmedetomidine. Ophthalmic examinations and multimodal corneal imaging were also performed at 1, 2, 4, 6, 8 and 12 months after initiating treatment with netarsudil or vehicle control. Additionally, patients received an ophthalmic examination including IOP measurement 1 week after starting the treatment to assess for potential adverse reactions.

### Clinical response criteria

Eyes were classified as progressed, stable or improved using any of the following response criteria when compared with baseline: (1) eyes were considered improved if they demonstrated a > 10% increase in ECD, a > 10% decrease of cornea affected by edema, and/or a > 20% decrease in CCT; (2) eyes were considered progressed if they demonstrated a > 10% decrease in ECD, a > 10% increase of cornea affected by edema, and/or a > 20% increase in CCT; (3) eyes were classified as stable if they did not meet criteria for improved nor progressed disease. The threshold for each criteria were defined by previous publications^23,32,40^. A significant decrease in CCT was deemed to be below 20% from the previous value taking into consideration that some patients would receive sodium chloride hypertonic ophthalmic ointment that might influence CCT values.

The percentage of change for eyes in the vehicle control group was calculated in two steps: difference comparing the 4-month and baseline exams (period when dogs received vehicle control or netarsudil) and difference comparing the 8-month and the 4-month exams (period when all dogs received topical netarsudil).

### Comparison with topical ripasudil

Lastly, a comparison was conducted between the efficacies of topical netarsudil applied BID and topical ripasudil 0.4% applied QID from our previously published study^24^. Any eye with 100% of the cornea affected by edema at the start of the clinical trial or less than 6 months of treatment were excluded. A total of 17 eyes from 11 dogs treated with ripasudil and 11 eyes from 9 dogs treated with netarsudil were included. Clinical progression was evaluated using the aforementioned clinical response criteria.

### Statistical analysis

Differences in CCT, percentage of the cornea affected by edema, and ECD across timepoints were evaluated by repeated measured analysis of variance (ANOVA) or a Friedman test for normally and non-normally distributed data, respectively. Normality was determined for each data set by the Shapiro Wilk test for normality. Normally distributed data were expressed as mean ± standard deviation (SD) and non-normally distributed data as median and IQR. Post-hoc tests were performed using Wilcoxon signed ranks. Clinical progression was represented using Kaplan–Meier curves for the netarsudil and vehicle control group, in which more than 10% of increase of corneal edema between timepoints was considered progression. Similarly, clinical progression of patients receiving netarsudil or ripasudil for 6 months were also compared using Kaplan–Meier curves. Log-rank test was employed to evaluate differences between curves. Fisher exact test was employed to compare proportions of eyes with progression, stabilization or improvement. For all statistical analysis, the data from each eye included in each group were considered individually. The statistical analysis was carried out in GraphPad Prism version 9.3.1 and VassarStats^41^.

### Supplementary Information

Supplementary Information.

## Data Availability

The datasets used and/or analyzed during the current study are not publicly available but will be made available from the corresponding author upon request.
