# Supplementary material for: Topical netarsudil for the treatment of primary corneal endothelial degeneration in dogs
Source: Sci Rep. 2024 Mar 14;14:6238. doi: 10.1038/s41598-024-56084-4 (PMC10940293; doi:10.1038/s41598-024-56084-4)
Supplement: Supplementary file 1 — Supplementary Information. [file 41598_2024_56084_MOESM1_ESM.docx]

Supplemental material

**A**

**B**

**C**

**D**

**Figure S1. Nasal, temporal, superior and inferior peripheral corneal thickness measured with USP did not differ between netarsudil or vehicle control groups or over time.** No significant differences were observed in corneal thickness measured with USP in the nasal (A), temporal (B), superior (C), and inferior (D) peripheral cornea neither between timepoints (P > 0.05), between patients on the netarsudil group and on the vehicle control group neither at baseline (P > 0.05), nor at any time point (P > 0.05). Two-way repeated measures ANOVA. Depicted are the mean and the standard deviation. Netarsudil group: baseline, 1, 2 and 4 months: 12 eyes. Six and 8 months: 11 eyes. Twelve months: 6 eyes. Vehicle control group: baseline, 1, 2 and 4 months: 14 eyes. Six months: 13 eyes. Eight months: 12 eyes. Twelve months: 5 eyes.

**Figure S2. Endothelial cell density did not differ over time or between groups using applanation IVCM.**  At baseline, no significant differences were observed in ECD between the vehicle control group (1772 ± 379 cells/mm^2^) and the netarsudil group (1689 ± 418 cells/mm^2^, *P* = 0.99). No significant differences were observed in ECD values measured with applanation IVCM neither between timepoints (P > 0.05), or between the netarsudil group and the vehicle control group at any time point (P > 0.05). Two-way repeated measures ANOVA. Depicted are the mean and the standard deviation. Netarsudil group: baseline, 1, 2 and 4 months: 12 eyes. Six and 8 months: 11 eyes. Vehicle control group: baseline, 1, 2 and 4 months: 14 eyes. Six months: 13 eyes. Eight months: 12 eyes.

Table S1. Summary of patient demographics, diagnosed with either corneal endothelial dystrophy (CED) or age-related endothelial degeneration, use of 5% saline (NaCl) ophthalmic ointment, and adverse reactions of patients included in our clinical trial.

|  | Group | Eyes In Study | Breed | Age | Sex | Diagnosis | Use of 5% NaCl Ointment | Adverse Reactions |
| --- | --- | --- | --- | --- | --- | --- | --- | --- |
| 1 | Netarsudil | 2 | Boston terrier | 11.3 | M | CED | Yes | Conjunctival hyperemia |
| 2 | Netarsudil | 2 | Jack Russell terrier | 10.2 | F | CED | No | Conjunctival hyperemia, unilateral reticulated intraepithelial bullae |
| 3 | Netarsudil | 1 | Chihuahua mix | 11.1 | M | CED | Yes | Conjunctival hyperemia, decreased IOP |
| 4 | Netarsudil | 1 | Boston terrier | 9.8 | F | CED | Yes | Conjunctival hyperemia |
| 5 | Netarsudil | 1 | Chihuahua mix | 11 | F | CED | Yes | Conjunctival hyperemia |
| 6 | Netarsudil | 1 | Chihuahua mix | 10.7 | F | CED | Yes | Conjunctival hyperemia |
| 7 | Netarsudil | 1 | Basset hound | 12.26 | F | Age-related endothelial degeneration | No | Conjunctival hyperemia |
| 8 | Netarsudil | 1 | Boston terrier | 12.3 | F | CED | Yes | Conjunctival hyperemia |
| 9 | Netarsudil | 1 | Boxer | 9.6 | M | Age-related endothelial degeneration | Yes | Conjunctival hyperemia |
| 10 | Netarsudil | 1 | Schipperke | 10.8 | M | Age-related endothelial degeneration | Yes | Conjunctival hyperemia |
| 11 | Vehicle control | 2 | Jack Russell terrier | 6.8 | M | CED | No | Conjunctival hyperemia, bilateral dry eye |
| 12 | Vehicle control | 2 | Jack Russell terrier | 7.2 | F | CED | No | Conjunctival hyperemia |
| 13 | Vehicle control | 1 | Boston terrier | 9.6 | F | CED | Yes | Conjunctival hyperemia, unilateral dry eye |
| 14 | Vehicle control | 1 | Labrador retriever | 11.7 | M | Age-related endothelial degeneration | Yes | Conjunctival hyperemia |
| 15 | Vehicle control | 1 | Boston terrier | 14.1 | F | CED | Yes | Conjunctival hyperemia |
| 16 | Vehicle control | 1 | Standard poodle | 11.1 | M | Age-related endothelial degeneration | No | Conjunctival hyperemia |
| 17 | Vehicle control | 1 | Shih tzu mix | 13.2 | F | Age-related endothelial degeneration | Yes | Conjunctival hyperemia |
| 18 | Vehicle control | 1 | Boston terrier | 9.9 | F | CED | Yes | Conjunctival hyperemia |
| 19 | Vehicle control | 1 | Boxer | 9.4 | F | Age-related endothelial degeneration | Yes | Conjunctival hyperemia |
| `20 | Vehicle control | 2 | Boston terrier | 8.4 | M | CED | No | Conjunctival hyperemia |
| 21 | Vehicle control | 1 | Brittany | 12 | F | Age-related endothelial degeneration | Yes | Conjunctival hyperemia |
